# The crosstalk between polycystic ovary syndrome and Hashimoto thyroiditis: a systematic review and meta-analysis

**DOI:** 10.3389/fendo.2026.1809000

**Published:** 2026-08-07

**Authors:** Yazeed Albalawi, Hyder Mirghani

**Affiliations:** 1 Obstetrics and Gynecology, Faculty of Medicine, University of Tabuk, Tabuk, Saudi Arabia; 2 Internal Medicine and Endocrinology, Faculty of Medicine, University of Tabuk, Tabuk, Saudi Arabia

**Keywords:** dyslipidemia, Hashimoto thyroiditis, insulin resistance, lipid profile, polycystic ovary syndrome

## Abstract

**Background:**

Polycystic ovary syndrome (PCOS) and Hashimoto thyroiditis (HT) are endocrine disorders; when they co-exist, they exacerbate each other’s serious consequences. Literature is scarce on these important diseases. This meta-analysis aimed to assess the prevalence of HT in both patients with PCOS and control subjects, as well as insulin resistance (IR) and lipid profile in patients with PCOS with and without HT.

**Methods:**

We systematically searched PubMed, Embase, Web of Science, and Google Scholar from inception to February 2026 using combinations of keywords and MeSH terms related to polycystic ovary syndrome, Hashimoto thyroiditis, autoimmune thyroid disease, insulin resistance, and metabolic outcomes. We retrieved 209 articles, of which 38 full texts were screened, and 22 studies were included in the meta-analysis.

**Results:**

HT was higher in patients with PCOS compared to control subjects (OR = 2.69, 95% CI: 1.63 to 4.42), and women with PCOS and HT exhibited higher HOMA-IR than women with PCOS alone (MD = −0.92, 95% CI: −1.50 to −0.35). However, the TC, LDL, HDL, and TG were similar in patients with PCOS with and without HT (MD = −0.61, 95% CI: −1.40 to 0.18; MD = −0.49, 95% CI: −1.69 to 0.72; MD = 0.01, 95% CI: −0.09 to 0.07; and MD = −0.06, 95% CI: −0.35 to 0.23, respectively).

**Conclusion:**

HT was more prevalent among women with PCOS than in controls. Women with PCOS and concomitant HT had higher IR than women with PCOS alone. However, the TC, LDL, HDL, and TG were similar in patients with PCOS with and without HT. Prospective studies with long follow-up are needed.

## Introduction

Polycystic ovary syndrome (PCOS) is a common disease with a prevalence of 9.2% worldwide. The syndrome is the most common metabolic disorder of women of reproductive age. Genetic predisposition and environmental and lifestyle factors are to blame (1). PCOS is a common and multifaceted endocrine condition; more than one in five (21%) of women of reproductive age are affected (2). The syndrome is defined by a constellation of clinical features (hyperandrogenism, irregular or absent ovulation, and characteristic polycystic ovarian morphology) that collectively contribute to a wide range of reproductive, metabolic, and psychological disturbances (3). Importantly, PCOS is associated with an increased susceptibility to long-term health complications such as type 2 diabetes mellitus (T2DM), cardiovascular disease, endometrial carcinoma, infertility, and adverse pregnancy outcomes. The diagnosis could be challenging due to the need for specific tests, the high variability of clinical features, and the need for a transvaginal ultrasound (4). The burden of PCOS is significant in the mother, particularly in relation to pregnancy outcomes, including eclampsia, labor induction, and miscarriage. Notably, children with a mother with PCOS are at a higher risk of diabetes mellitus and obesity [5]. PCOS is on the rise with more severe phenotypes possibly reflecting a higher prevalence of infertility, diabetes, and hyperandrogenism (5).

The low progesterone levels in PCOS initiate an immune response leading to high estrogen and autoantibodies, including antinuclear antibodies, anti-ovarian antibodies, and antithyroid antibodies. Therefore, PCOS could be associated with autoimmune disorders, including Hashimoto thyroiditis (HT) (6). On the other hand, a large study conducted in Asia involving 1,808 infertile women found that no excess positive thyroid peroxidase antibodies (TPO Ab) were found in women with PCOS compared to their counterparts without the syndrome (7).

Hashimoto thyroiditis affects 7.5% globally, with Africa bearing the highest prevalence compared to South America, Europe, and North America (14.2%, 7.8%, and 5.8%, respectively). HT is associated with cardiovascular disease and malignancy (8). When PCOS and HT coexist, they are linked to an increased likelihood of serious metabolic and reproductive complications and malignancies. Genetic influences play a significant role in the development of both PCOS and HT, and multiple shared susceptibility genes associated with an elevated risk of these conditions have been identified (9).

An interesting study found that thyroid antibodies are more prevalent in type A and B phenotypes of PCOS compared to types C and D. Plausible explanations could be the greater imbalance between estrogen and progesterone in different phenotypes (10).

Oxidative stress, immune response, and pro-inflammation interplay are suggested to play a major role in the coexistence of these two major endocrine disorders. When these two conditions coexist, they exacerbate each other’s serious consequences (11).

Evidence shows that continuous exposure to autoantibodies in patients with PCOS and HT negatively affects ovarian function (12). The primary objective of this meta-analysis was to assess the prevalence of HT in both patients with PCOS and control subjects. The secondary objective was to assess insulin resistance (HOMA-IR) and lipid profile in patients with PCOS with and without HT.

## Subjects and methods

This systematic review and meta-analysis was conducted from January 2025 to February 2026 to assess HT in the PCOS and control groups. In addition, we investigated the lipid profile and insulin resistance in women with PCOS with and without HT. The study was conducted with strict adherence to the Preferred Reporting Items for Systematic Reviews and Meta-Analyses (PRISMA) guidelines.

### Eligibility criteria

#### Study type

We included randomized controlled trials, cross-sectional studies, case–control studies, retrospective studies, and prospective studies. The studies must assess the rate of HT in both patients with PCOS and control subjects. HOMA-IR values and lipid profile were compared between women with PCOS and HT and those with PCOS alone.

### Exclusion criteria

Case reports, opinions, editorials, commentaries, and protocols without results were excluded.

### Participants

The participants in the study were patients with PCOS and control subjects.

### Outcome measures

#### Primary outcomes

The outcome measures were the rate of HT in PCOS patients and control subjects.

#### Secondary outcomes

These included insulin resistance and lipid profile in patients with PCOS with and without HT.

### Literature search

**Figure 1:**
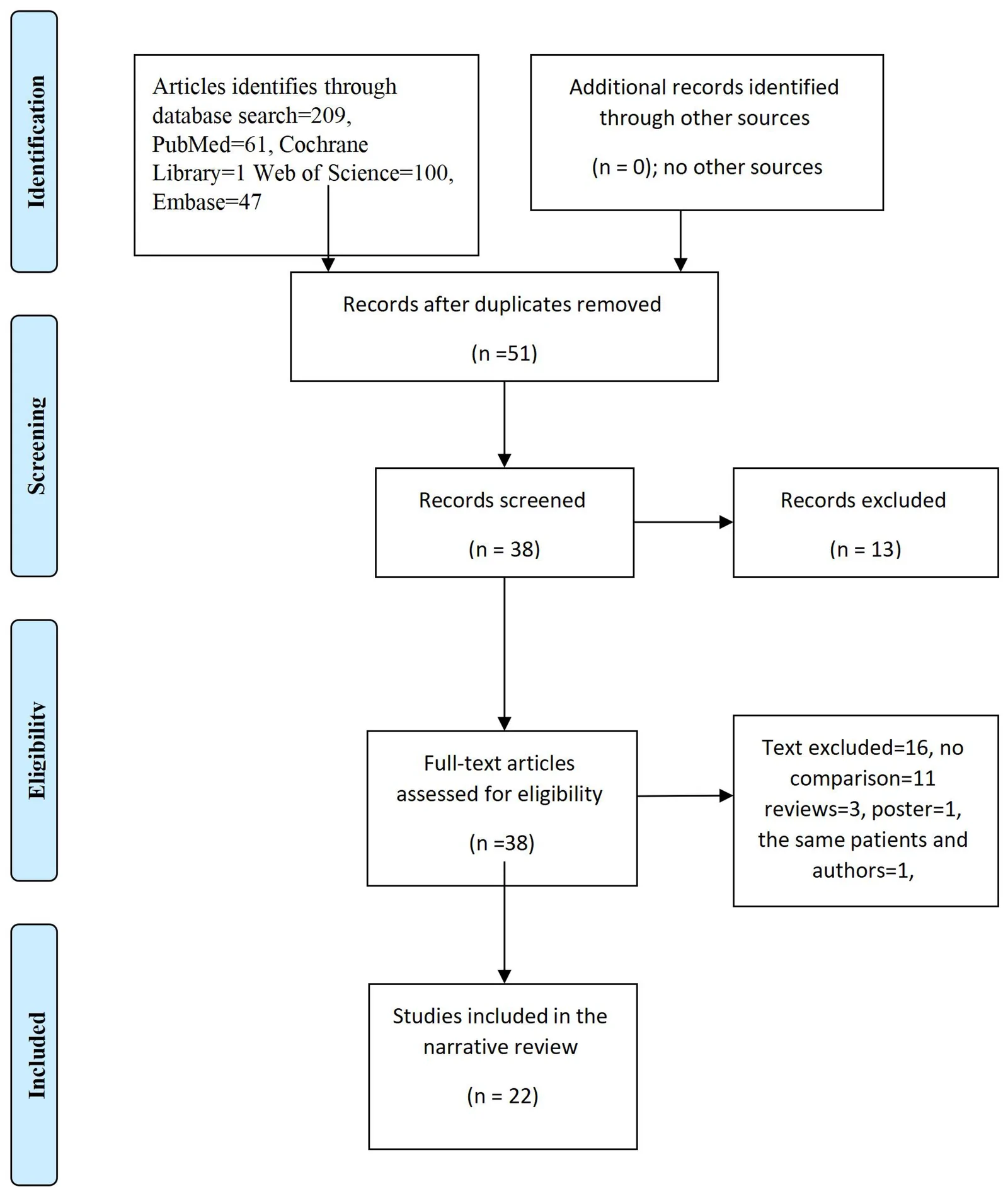
Prevalence of PCOS in patients with HT, insulin resistance, and dyslipidemia with PCOS with and without HT (the PRISMA chart).

Two authors (H. M. and Y. A.) conducted a comprehensive literature search in accordance with the PRISMA guidelines. A comprehensive literature search was conducted in PubMed, Embase, Web of Science, Cochrane Library, and Google Scholar using combinations of keywords and MeSH terms related to polycystic ovary syndrome, Hashimoto thyroiditis, autoimmune thyroid disease, insulin resistance, and metabolic outcomes. The search covered publications from database inception to February 2026. Only articles published in the English language were considered eligible for inclusion. A total of 209 records were identified through database searching. After removal of 158 duplicate records, 51 articles remained for title and abstract screening. Thirteen records were excluded during screening, leaving 38 articles for full-text assessment. Eleven full-text articles were excluded for predefined reasons, resulting in 27 eligible studies. Five additional studies were subsequently excluded because they were reviews (*n* = 3), conference abstracts (*n* = 1), or duplicate reports from the same population (*n* = 1), leaving 22 studies for inclusion in the final meta-analysis (**Figure 1**).

### Data extraction

First author name, country and year of publication, age in PCOS and controls, BMI in PCOS and control subjects, insulin resistance, and lipid profile in PCOS with and without HT were reported in tables and transferred manually to RevMan 5.4 for meta-analysis (**Tables 1**–**3**).

**Table 1:** Hashimoto thyroiditis in both patients with PCOS and control subjects.

Author	Country	PCOS/total	Controls/total	Study type	Diagnosis	HT/PCOS	HT/controls	Age/PCOS	Age/controls	BMI/PCOS	BMI/controls
Admaska et al., 2020 (13)	Poland	141	88	Cross-sectional	Rotterdam criteria	31	21	24.9 ± 1.13	24.56 ± 1.25	22.2 ± 0.85	23.7 ± 1.28
Al-Saab et al., 2014 (14)	Syria	56	30	Case–control	Rotterdam criteria	11	1	23.8 ± 5.6	28.9 ± 5.8	24.9 ± 5.9	23.9 ± 2.9
Arduc et al., 2015 (15)	Turkey	86	60	Cross-sectional	Rotterdam criteria	19	3	25.6 ± 5.4	27.8 ± 5.9	27.9 ± 6.3	25.1 ± 5.0
Arora et al., 2016 (16)	India	55	51	Cross-sectional	Rotterdam criteria	21	8	23.27 ± 3.2	22.8 ± 4.4	Not reported	Not reported
Calvar et al. (17)	Argentina	142	52	Case–control	Rotterdam criteria	27	7	25.0 ± 5.2	27.0 ± 6.1	26.4 ± 5.3	25.8 ± 4.9
Duran et al., 2015 (18)	Turkey	73	60	Cross-sectional	Rotterdam criteria	23	14	26.1 ± 4.9	24.2 ± 4.8	27.45 ± 5.73	22.55 ± 3.78
Ganie et al., 2010 (19)	India	175	46	Case–control	Rotterdam criteria	82	20	15.7 ± 2.8	14.72 ± 1.57	29.89 ± 4.04	27.03 ± 4.92
Garelli et al., 2013 (20)	Italy	113	100	Cross-sectional	Rotterdam criteria	30	8	26.4 ± 5.1	28.1 ± 4.9	26.1 ± 5.7	24.8 ± 4.3
Ho et al., 2020 (21)	china	1332	2664	Cross-sectional	Rotterdam criteria	19	16	43.7 ± 14.4	45.1 ± 16.3	Not reported	Not reported
Ho et al., 2020 (22)	Taiwan	1332	26924	Cross-sectional	Rotterdam criteria	119	280	43.7 ± 16.5	42.3 ± 14.5	Not reported	Not reported
Janssden et al., 2004 (23)	Germany	175	168	Prospective		47	14	28.4± 6.5	29.8 ± 7.4	30.0 ± 7.9	25.5 ± 7.1
Kachuei et al., 2012 (24)	Iran	72	90	Case–control	Rotterdam criteria	22	25	26.1 ± 5.3	27.4 ± 4.8	27.2 ± 5.4	25.1 ± 4.6
Karakose et al., 2017 (25)	Turkey	97	71	Cross-sectional	Rotterdam criteria	39	11	21.1 ± 6	24.4 ± 4.5	27.5 ± 6	23.4 ± 5
Kim et al., 2022 (26)	Korea	104	238	Case–control	Rotterdam criteria	5	18	Not reported	Not reported	Not reported	Not reported
Novais et al., 2015 (27)	Brazil	65	65	Cross-sectional	Rotterdam criteria	28	17	26.3 ± 4.4	27.1 ± 5.0	27.5 ± 5.6	24.6 ± 4.1
Petrikova et al., 2012 (28)	Slovak Republic	64	68	Cross-sectional	Rotterdam criteria	12	7	30.23 ± 6.7	30.23 ± 6.7	28.08 + 6.91	21.31 + 3.05
Sinha et al., 2013 (29)	India	80	80	Cross-sectional	Rotterdam criteria	18	1	22.7 ± 5.30	24.3 ± 5.69	24.68 ± 3.07	23.55 ± 3.02
Skrzynska et al., 2022 (30)	Poland	80	64	Cross-sectional	Rotterdam criteria updates	18	9	16.54 ± 1	16.71 ± 0.63	24.6 ± 4.16	22.8 ± 3.27
Yu and Wang 2016 (31)	China	100	100	Cross-sectional	Rotterdam	25	2	27.4 ± 5.4	23.3 ± 4.1	22.2 ± 0.85	29.2 ± 5.1
Jia et al., 2023 (32)	China	49	164	Cross-sectional	Rotterdam criteria	Not reported	Not reported	Not reported	Not reported	Not reported	Not reported
Serin et al., 2021 (12)	Turkey	46	46	Prospective	Rotterdam criteria	Not reported	Not reported	Not reported	Not reported	Not reported	Not reported
Zhao et al., 2021 (33)	China	52	112	Retrospective	Rotterdam criteria	Not reported	Not reported	Not reported	Not reported	Not reported	Not reported

**Table 2:** Insulin resistance and lipid profile in patients with PCOS with and without Hashimoto thyroiditis.

Author	HT	No HT	IR	IR	HDL	HDL	TG	TG	TC	TC	LDL	LDL
Duran et al., 2015 (18)	73	60	2.96 ± 2.11	1.77 ± 0.83	Not reported	Not reported	Not reported	Not reported	Not reported	Not reported	Not reported	Not reported
Ganie et al., 2010 (19)	175	46	4.4 ± 4.2	2.3 ± 2.7	1.01 ± 0.12	1.08 ± 0.17	1.57 ± 0.32	1.22 ± 0.23	5.41 ± 0.70	3.85 ± 0.64	2.90 ± 0.44	1.08 ± 0.17
Janssen et al., 2004 (23)	47	128	5.8 ± 5.3	4.4 ± 3.8	Not reported	Not reported	Not reported	Not reported	Not reported	Not reported	Not reported	Not reported
Jia et al., 2023 (32)	49	164	2.76 ± 1.29	2.30 ± 1.28	1.50 ± 0.40	1.43 ± 0.52	1.28 ± 0.83	1.34 ± 1.05	4.88 ± 1.31	4.38 ± 1.14	2.71 ± 0.65	2.75 ± 0.77
Serin et al., 2021 (12)	46	46	4.6 ± 6.6	5.3 ± 9.2	1.23 ± 0.31	1.19 ± 0.25	1.42 ± 0.94	1.48 ± 0.79	4.58 ± 0.98	4.49 ± 0.87	2.75 ± 0.93	2.66 ± 0.74
Zhao et al., 2021 (33)	52	112	3.01 ± 2.16	2.69 ± 2.21	1.28 ± 0.25	1.23 ± 0.27	1.31 ± 0.89	1.4 ± 1.01	4.69 ± 0.98	4.44 ± 1.05	2.60 ± 0.94	2.54 ± 0.83

**Table 3:** Risk of bias of the included observational studies.

Author	Selection	Comparability	Outcome	Total score
Admaska et al., 2020 (13)	4	1	3	8
Al-Saab et al., 2014 (14)	4	2	3	9
Arduc et al., 2015 (15)	4	2	3	9
Arora et al., 2016 (16)	4	1	3	8
Calvar et al. (17)	4	2	3	9
Duran et al., 2015 (18)	4	1	3	8
Ganie et al., 2010 (19)	4	2	3	9
Garelli et al., 2013 (20)	4	1	3	8
Ho et al., 2020 (21)	4	1	3	8
Ho et al., 2020 (22)	4	1	3	8
Janssden et al., 2004 (23)	4	1	3	8
Kachuei et al., 2012 (24)	4	2	3	9
Karakose et al., 2017 (25)	4	2	3	9
Kim et al., 2022 (26)	4	1	3	8
Novais et al., 2015 (27)	4	1	3	8
Petrikova et al., 2012 (28)	4	2	3	9
Sinha et al., 2013 (29)	4	2	3	9
Skrzynska et al., 2022 (30)	4	2	3	9
Yu and Wang 2016 (31)	4	2	3	9
Jia et al., 2023 (32)	4	2	3	9
Serin et al., 2021 (12)	4	1	3	8
Zhao et al., 2021 (33)	4	2	3	9

### PCOS diagnosis

PCOS was diagnosed based on the Rotterdam diagnostic criteria and the international collaboration of pediatric endocrine and other societies (34, 35).

### Risk of bias assessment

Methodological quality was independently assessed by two reviewers using the Newcastle–Ottawa Scale (NOS) for observational studies (36). Any discrepancies were resolved by consensus (**Table 4**).

**Table 4:** Analysis of the quality of evidence by Grading of Recommendations, Assessment, Development, and Evaluation (GRADE).

Outcome	Number of studies	Study design	Risk of bias	Inconsistency	Indirectness	Imprecision n	Other consideration	Certainty of evidence
Prevalence of Hashimoto thyroiditis in PCOS	19	Cross-sectional = 15, case–control = 2, prospective = 2	Moderate	Serious (I2 = 88%)	Not serious	Not serious	None	Low
Insulin resistance in patients with PCOS with and without HT	6		Moderate	Serious (I2 = 66%)	Not serious	Not serious	None	Low
Total cholesterol in patients with PCOS with and without HT	4		Moderate	Serious (I2 = 96%)	Not serious	Not serious	None	Low
LDL in patients with PCOS with and without HT	4		Moderate	Serious (I2 = 99%)	Not serious	Not serious	None	Low
HDL in patients with PCOS with and without HT	4		Moderate	Serious (I2 = 66%)	Not serious	Not serious	None	Low
TG in patients with PCOS with and without HT	4		Moderate	Serious (I2 = 83%)	Not serious	Not serious	None	Low

### Data analysis

The Cochrane system for systematic review (RevMan, version 5.4.1, Oxford, United Kingdom) was used for data analysis. The data for the prevalence of HT in patients with PCOS were dichotomous, and the data for insulin resistance (HOMA-IR) and lipid profile were continuous; the random-effects model was used due to significant heterogeneity. In this meta-analysis, mean differences were calculated as PCOS alone minus PCOS+HT. Therefore, negative values indicate higher measurements among women with both PCOS and HT. The *I*^2^ was used to evaluate heterogeneity among studies (heterogeneity ≤25% was considered low, ≥50% was considered substantial, and the random effect was used). We investigated the prevalence of HT in patients with PCOS. In addition, HOMA-IR values were compared between women with PCOS and HT and those with PCOS alone, and the lipid profile in patients with PCOS with and without HT was assessed. The continuous data were evaluated as mean difference (MD), and the dichotomous data were reported as odds ratios using forest plots. Funnel plots were used in the analysis with ≥10 studies for heterogeneity, followed by a subanalysis in which we assessed the case–control and cross-sectional studies separately to locate the source of heterogeneity. The chi-square, *Z*, and standard difference were estimated to assess the prevalence of HT in both patients with PCOS and control subjects, as well as insulin resistance and lipid profile in patients with PCOS with and without HT. We adopted a 95% confidence interval, and *P*-values <0.05 were considered significant.

## Results

### Characteristics of the included studies

In the present meta-analysis, 22 studies (12–33) were included; 15 studies were from Asia, 5 studies were published in Europe, and 2 were conducted in South America. Fourteen studies were cross-sectional, 5 were case–control studies, 2 were prospective, and 1 was a retrospective study. The patients’ age ranged from 14.72 ± 1.57 to 45.1 ± 16.3 years, and their BMI ranged from 22.2 ± 0.85 to 30.0 ± 7.9. The Rotterdam criteria were used in the majority of patients.

### The prevalence of HT in both patients with PCOS and control subjects

In this meta-analysis, we included 19 observational studies to assess the prevalence of HT in both patients with PCOS and control subjects. A total of 4,342 individuals with PCOS and 31,019 controls were included. Overall, the analysis indicates a statistically significantly higher HT in patients with PCOS compared to their counterparts without the syndrome (OR = 2.69, 95% CI: 1.63 to 4.42), with substantial between-study heterogeneity (*I*^2^ = 88%, *τ*^2^ = 0.99; *χ*^2^ = 153.73, *P* < 0.00001). The pooled estimate, calculated using a random-effect model, is shown as a diamond.

### Subgroup analysis

#### Cross-sectional studies

A statistically significantly higher HT in patients with PCOS compared to their counterparts without the syndrome was observed when including only cross-sectional studies (OR = 3.35, 95% CI: 1.86 to 6.02), with substantial between-study heterogeneity (*I*^2^ = 88%, *τ*^2^ = 0.93; *χ*^2^ = 99.12, *P* < 0.00001). Following sensitivity analysis, HT remained significantly higher in patients with PCOS (OR = 2.83, 95% CI: 2.11–3.80; *Z* = 6.94, *P* < 0.00001), with no evidence of between-study heterogeneity (*I*^2^ = 0%).

#### Case–control studies

No significant statistical difference was found regarding the prevalence of HT in patients with PCOS compared to their counterparts without the syndrome when including only case–control studies (OR = 1.22, 95% CI: 0.84 to 1.76), with no between-study heterogeneity (*I*^2^ = 15%; *χ*^2^ = 4.69, *P* = 0.29). The results remained not significant following sensitivity analysis (OR = 1.10, 95% CI: 0.75–1.60; *Z* = 0.48, *P* = 0.63), with no evidence of between-study heterogeneity (*I*^2^ = 0%).

### Geographical and age variations

**Figure 2:**
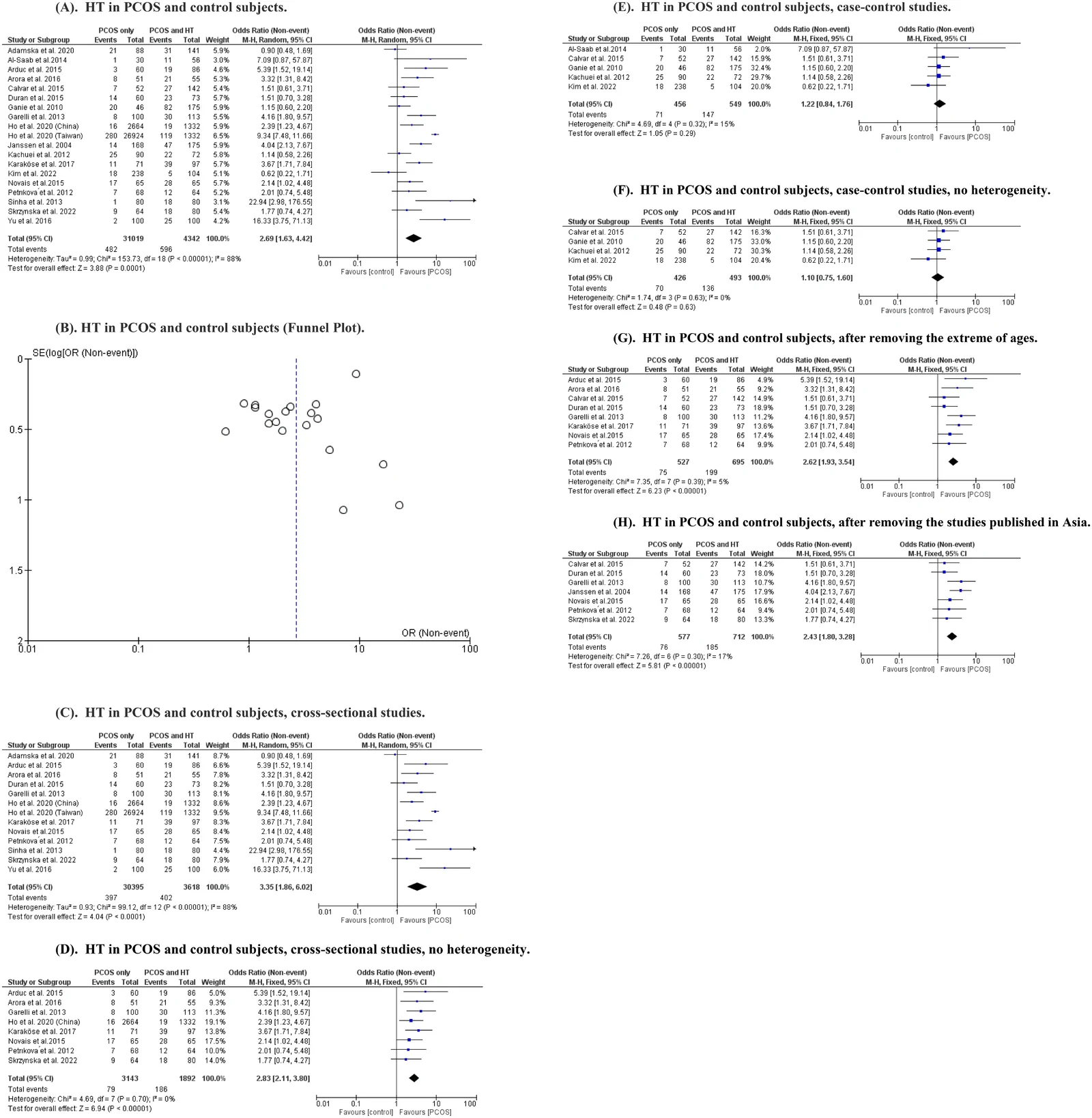
**(A)** HT in patients with PCOS and control subjects. **(B)** HT in patients with PCOS and control subjects (funnel plot). **(C)** HT in patients with PCOS and control subjects (cross-sectional studies). **(D)** HT in patients with PCOS and control subjects (cross-sectional studies, no heterogeneity). **(E)** HT in patients with PCOS and control subjects (case–control studies). **(F)** HT in patients with PCOS and control subjects (case–control studies, no heterogeneity). **(G)** HT in patients with PCOS and control subjects, after removing the extreme of ages. **(H)** HT in patients with PCOS and control subjects after removing the studies published in Asia.

We conducted a sensitivity analysis after removing the extreme of ages and studies published in Asia. HT remained significantly higher in patients with PCOS (OR = 2.62, 95% CI: 1.93–3.54; *Z* = 6.23, *P* < 0.00001), with no significant between-study heterogeneity (*I*^2^ = 5%), and control subjects (OR = 2.43, 95% CI: 1.80–3.28; *Z* = 5.81, *P* < 0.00001), with no significant between-study heterogeneity (*I*^2^ = 17%) (**Figures 2A–H**, **Tables 5**, **6**).

**Table 5:** The effects of different studies on heterogeneity in the HT prevalence arm, removing studies one by one.

Author	Country	Effect on heterogeneity
Admaska et al., 2020 (13)	Poland	1% decrease
Al-Saab et al., 2014 (14)	Syria	1% increase
Arduc et al., 2015 (15)	Turkey	1% increase
Arora et al., 2016 (16)	India	1% increase
Calvar et al. (17)	Argentina	1% increase
Duran et al., 2015 (18)	Turkey	No change
Ganie et al., 2010 (19)	India	No change
Garelli et al., 2013 (20)	Italy	1% increase
Ho et al., 2020 (21)	China	1% increase
Ho et al., 2020 (22)	Taiwan	22% decrease
Janssden et al., 2004 (23)	Germany	1% increase
Kachuei et al., 2012 (24)	Iran	No change
Karakose et al., 2017 (25)	Turkey	1% increase
Kim et al., 2022 (26)	Korea	No change
Novais et al., 2015 (27)	Brazil	No change
Petrikova et al., 2012 (28)	Slovak Republic	1% increase
Sinha et al., 2013 (29)	India	No change
Skrzynska et al., 2022 (30)	Poland	1% increase
Yu and Wang 2016 (31)	China	1% increase

**Table 6:** The effects of different studies on heterogeneity in the insulin resistance arm, removing studies one by one.

Author	Country	Effect on heterogeneity
Ganie et al., 2010 (19)	India	22% decrease
Duran et al., 2015 (18)	Turkey	1% decrease
Janssden et al., 2004 (23)	Germany	6% increase
Jia et al., 2023 (32)	China	6% decrease
Serin et al., 2021 (12)	Turkey	5% increase
Zhao et al., 2021 (33)	China	3% increase

### Insulin resistance

**Figure 3:**
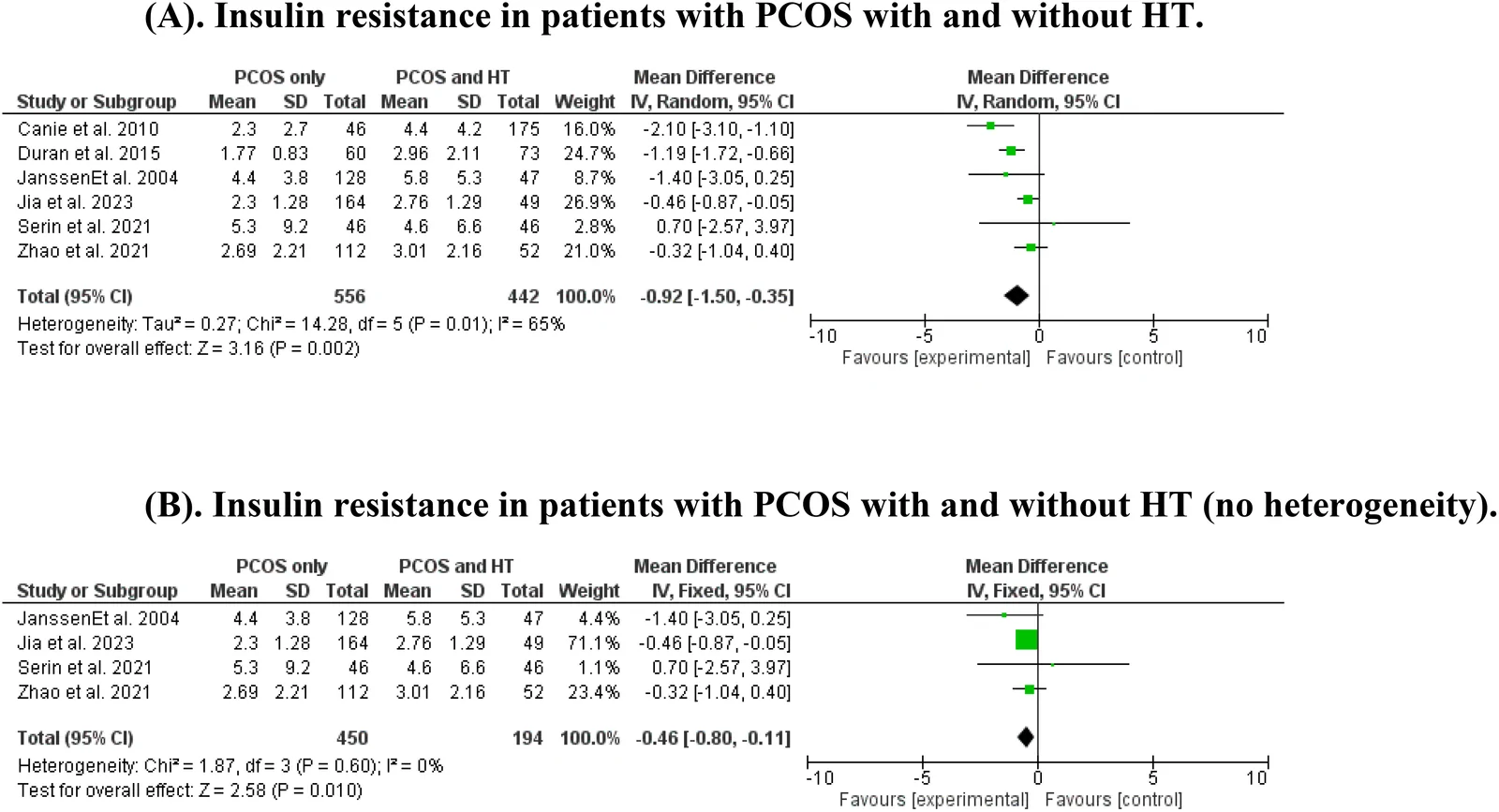
**(A)** Insulin resistance in patients with PCOS with and without HT. **(B)** Insulin resistance in patients with PCOS with and without HT (no heterogeneity).

In this meta-analysis, women with PCOS and concomitant HT had significantly higher HOMA-IR values than women with PCOS alone (MD = −0.92, 95% CI: −1.50 to −0.35, calculated as PCOS only minus PCOS+HT; *I*^2^ = 65%; *P*-value = 0.002, *df* = 5, and *Z* = 3.16). However, a significant heterogeneity was found (*I*^2^ = 65, *χ*^2^ = 14.28, and *P*-value for heterogeneity = 0.01). Sensitivity analyses, excluding studies contributing most to heterogeneity, yielded similar results (MD = −0.46, 95% CI: −0.80 to −0.11; *P*-value = 0.01, *df* = 3, *Z* = 2.58), and no significant heterogeneity was found (*I*^2^ = 0, *χ*^2^ = 1.87, *P*-value for heterogeneity = 0.60) (**Figures 3A, B**).

### Lipid profile

**Figure 4:**
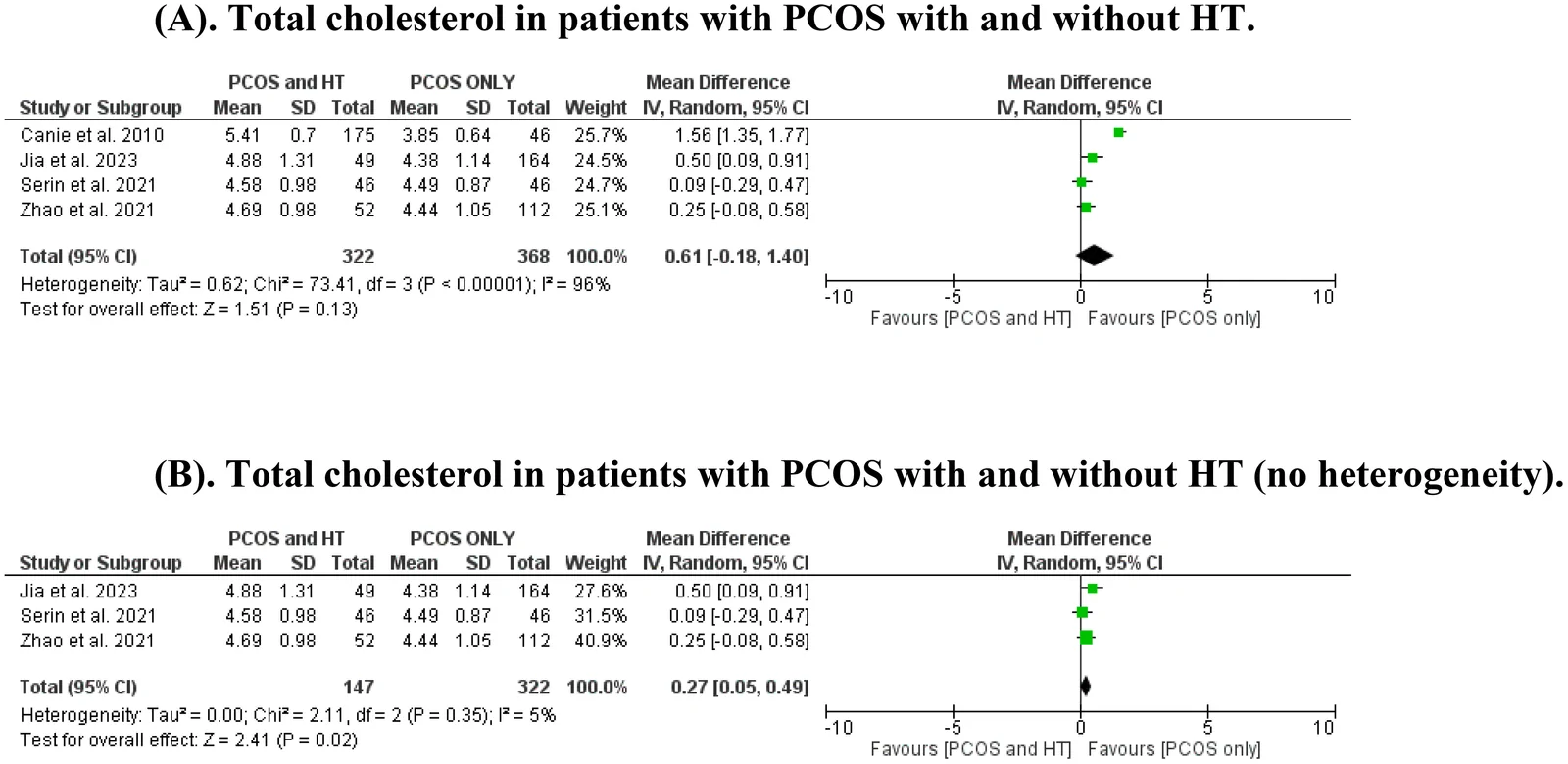
**(A)** Total cholesterol in patients with PCOS with and without HT. **(B)** Total cholesterol in patients with PCOS with and without HT (no heterogeneity).

Total cholesterol was not different in patients with PCOS and HT compared to those with PCOS only (MD = −0.61, 95% CI: −1.40 to 0.18; *P*-value = 0.13, *df* = 3, *Z* = 1.51). However, a significant heterogeneity was found (*I*^2^ = 96, *χ*^2^ = 73.41, and *P*-value for heterogeneity < 0.001). In a subgroup analysis with removal of studies with significant heterogeneity, total cholesterol was higher in patients with PCOS and HT (MD = −0.27, 95% CI: −0.48 to −0.06; *P*-value = 0.01, *df* = 2, *Z* = 2.48), and no significant heterogeneity was found (*I*^2^ = 5, *χ*^2^ = 2.11, and *P*-value for heterogeneity = 0.35) (**Figures 4A, B**).

**Figure 5:**
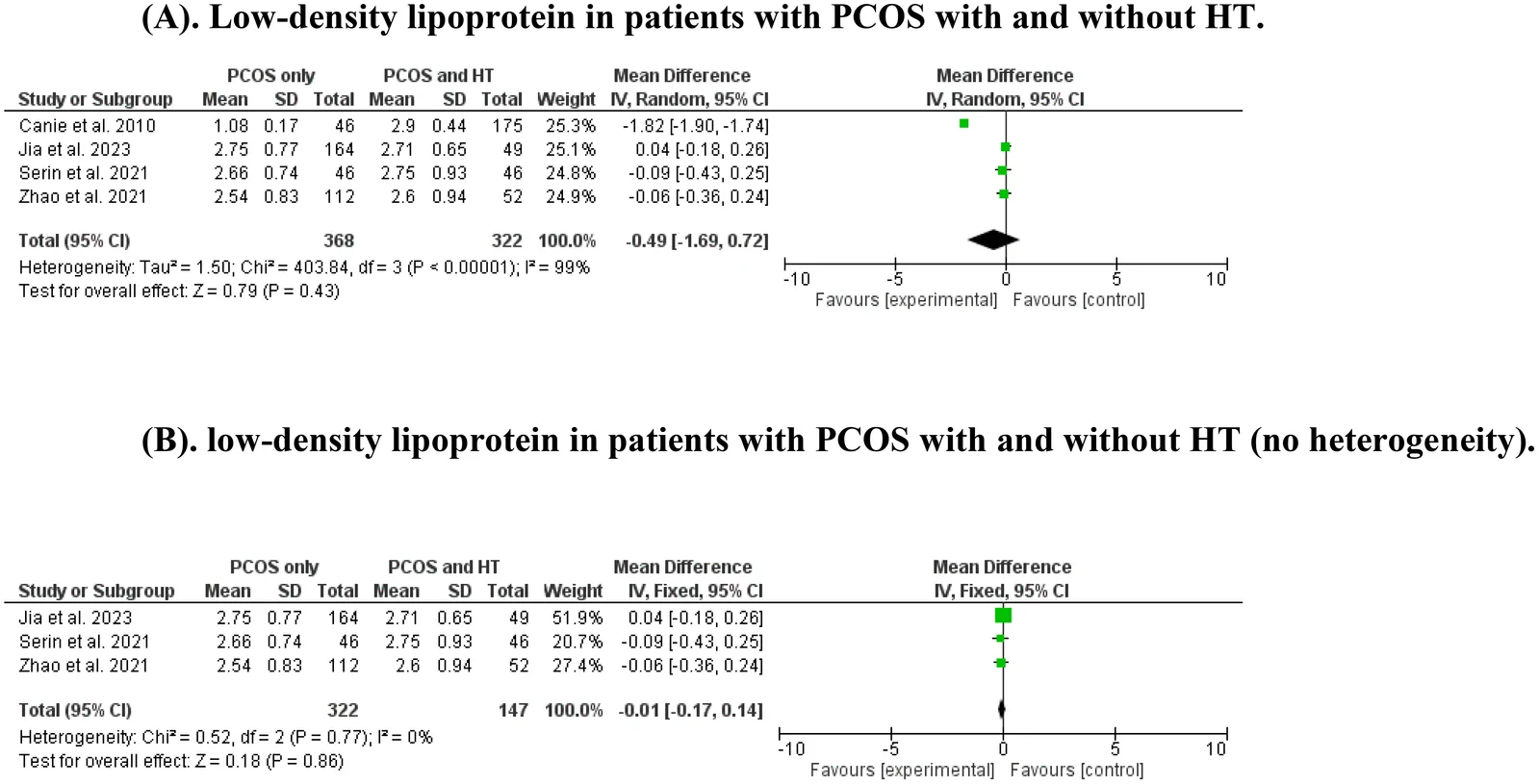
**(A)** Low-density lipoprotein in patients with PCOS with and without HT. **(B)** Low-density lipoprotein in patients with PCOS with and without HT (no heterogeneity).

LDL was not different in patients with PCOS and HT compared to those with PCOS only (MD = −0.49, 95% CI: −1.69 to 0.72; *P*-value = 0.43, *df* = 3, *Z* = 0.79). However, a significant heterogeneity was found (*I*^2^ = 99, *χ*^2^ = 403.84, and *P*-value for heterogeneity < 0.001). In a subgroup analysis with removal of studies with significant heterogeneity, LDL was not different in patients with PCOS and HT (MD = −0.01, 95% CI: −0.17 to 0.14; *P*-value, 0.86, *df* = 2, *Z* = 0.18), and no significant heterogeneity was found (*I*^2^ = 0, *χ*^2^ = 0.52, and P-value for heterogeneity = 0.77) (**Figures 5A, B**).

**Figure 6:**
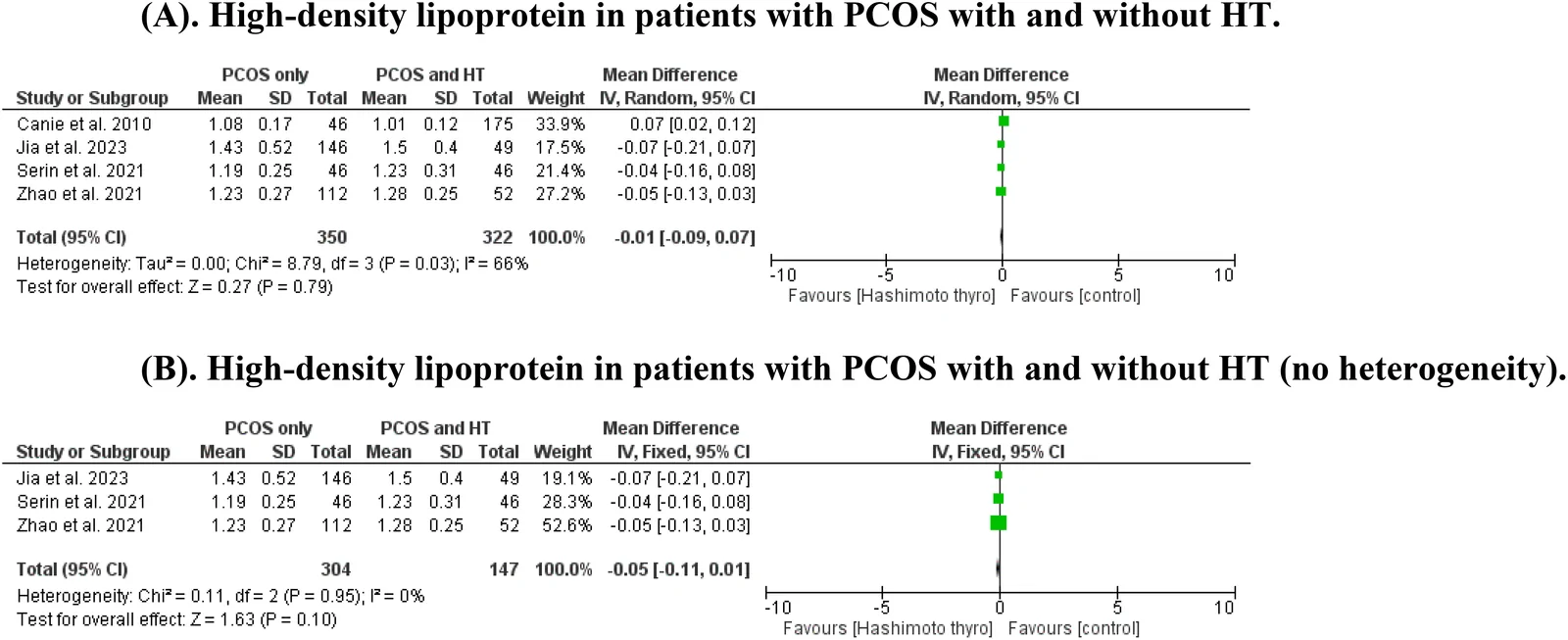
**(A)** High-density lipoprotein in patients with PCOS with and without HT. **(B)** High-density lipoprotein in patients with PCOS with and without HT (no heterogeneity).

HDL was not different in patients with PCOS and HT compared to those with PCOS only (MD = 0.01, 95% CI: −0.09 to 0.07; *P*-value = 0.79, *df* = 3, *Z* = 0.27). However, a significant heterogeneity was found (*I*^2^ = 66, *χ*^2^ = 8.79, and *P*-value for heterogeneity = 0.03). In a subgroup analysis with removal of studies with significant heterogeneity, HDL was not different in patients with PCOS and HT (MD = 0.05, 95% CI: –0.01 to 0.11; *P*-value = 0.10, *df* = 2, *Z* = 1.63), and no significant heterogeneity was found (*I*^2^ = 0, *χ*^2^ = 0.11, and *P*-value for heterogeneity = 0.95) (**Figures 6A, B**).

**Figure 7:**
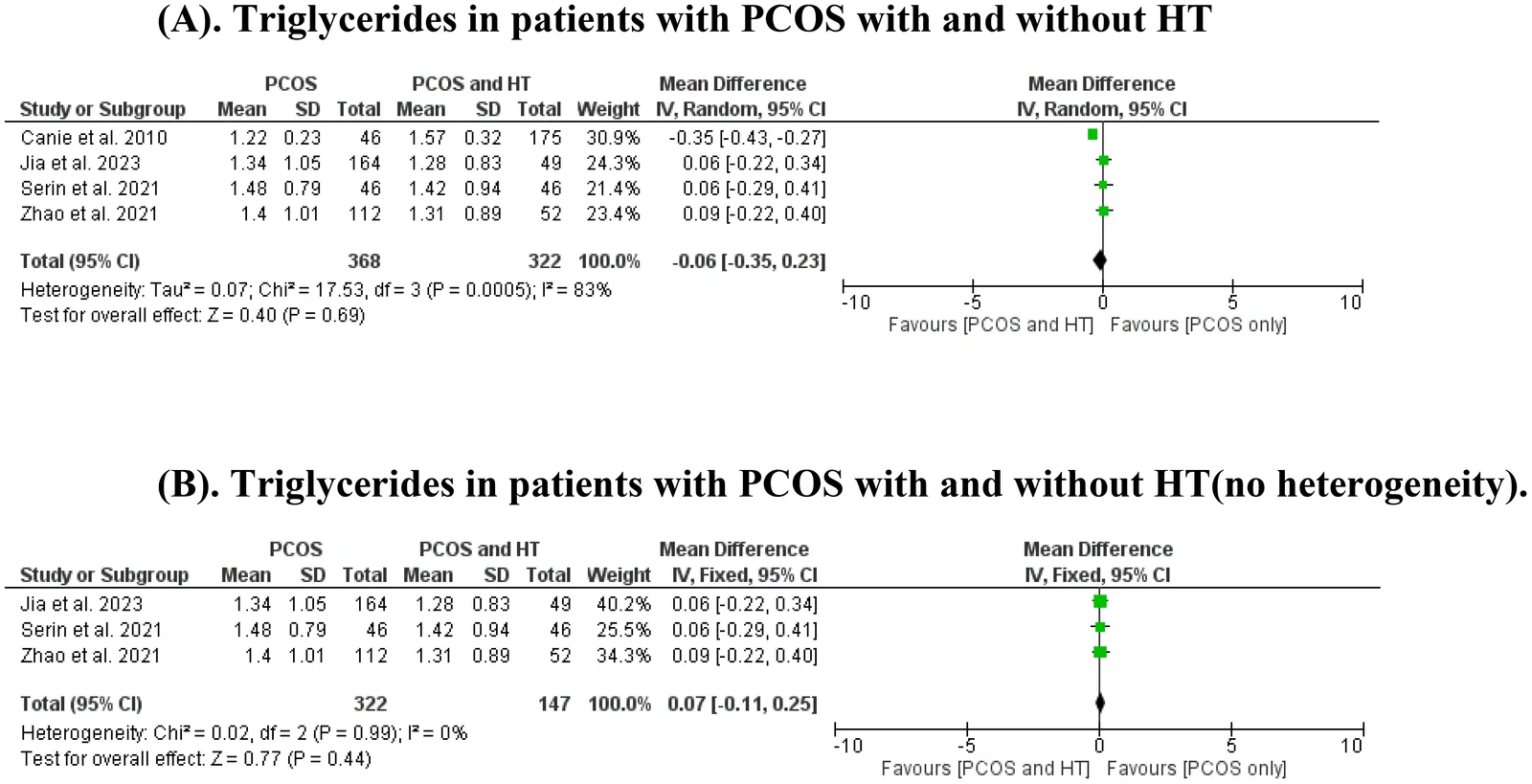
**(A)** Triglycerides in patients with PCOS with and without HT. **(B)** Triglycerides in patients with PCOS with and without HT (no heterogeneity).

Triglycerides were not different in patients with PCOS and HT compared to those with PCOS only (MD = −0.06, 95% CI: −0.35 to 0.23; *P*-value = 0.69, *df* = 3, *Z* = 0.40). However, a significant heterogeneity was found (*I*^2^ = 83, *χ*^2^ = 17.53, and *P*-value for heterogeneity = 0.0005). In a subgroup analysis with removal of studies with significant heterogeneity, triglycerides were not different in patients with PCOS and HT (MD = −0.07, 95% CI: −0.11 to 0.25; *P*-value = 0.44, *df* = 2, *Z* = 0.77), and no significant heterogeneity was found (*I*^2^ = 0, *χ*^2^ = 0.02, and *P*-value for heterogeneity = 0.99) (**Figures 7A, B**).

## Discussion

In this meta-analysis, the prevalence of HT was higher in patients with PCOS compared to controls (OR = 2.69, 95% CI: 1.63 to 4.42). Women with PCOS and concomitant HT had significantly higher HOMA-IR values than women with PCOS alone (MD = −0.92, 95% CI: −1.50 to −0.35). Mean differences were calculated as PCOS alone minus PCOS+HT. Therefore, negative values indicate higher measurements among women with both PCOS and HT. However, the TC, LDL, HDL, and TG were similar in patients with PCOS with and without HT (MD = −0.61, 95% CI: −1.40 to 0.18; MD = −0.49, 95% CI: −1.69 to 0.72; MD = 0.01, 95% CI: −0.09 to 0.07; and MD = −0.06, 95% CI: −0.35 to 0.23, respectively). A statistically significantly higher HT in patients with PCOS compared to their counterparts without the syndrome was observed when including only cross-sectional studies (OR = 3.35, 95% CI: 1.86 to 6.02). However, no significant statistical difference was found regarding the prevalence of HT in patients with PCOS compared to their counterparts without the syndrome when including only case–control studies (OR = 1.22, 95% CI: 0.84 to 1.76). In addition, the prevalence of HT was significantly higher in patients with PCOS compared to their counterparts without the syndrome when removing the extreme of ages (adolescents and middle-aged women) (OR = 2.62, 95% CI: 1.93–3.54; *Z* = 6.23 and OR = 2.43, 95% CI: 1.80–3.28; *Z* = 5.81, respectively).

The current findings were similar to those of Romitti et al. (37), who included 13 studies and found a higher prevalence of HT in patients with PCOS. Importantly, the authors assessed all autoimmune thyroid diseases in different geographical locations. Another meta-analysis (38) that included only six studies found a high prevalence of autoimmune thyroiditis in patients with PCOS compared to controls. Our findings were similar to those of Hu et al. (39), who included only 18 studies with overlap (40). In addition, the authors included a poster presentation (41), which significantly limited their results.

In this meta-analysis, we included the largest up-to-date studies. In addition, we assessed insulin resistance and lipid profile in patients with PCOS with and without HT.

HT is the most common autoimmune thyroid disorder and has been consistently associated with metabolic and reproductive abnormalities, including insulin resistance and PCOS. Increasing evidence suggests that this association is multifactorial, involving shared autoimmune mechanisms, hormonal dysregulation, metabolic alterations, and genetic susceptibility. A study conducted in Asia found that HT is associated with increasing ovarian volume and insulin resistance (42). Another plausible explanation is the shared mechanism of low-grade inflammation and increased inflammatory markers, including C-reactive protein, interleukin-6, and tumor necrosis factor-α (43). This inflammatory milieu may impair insulin signaling pathways, thereby contributing to insulin resistance. Moreover, autoimmune predisposition may explain the higher prevalence of thyroid autoantibodies in women with PCOS compared with the general population (44).

The pathogenesis of PCOS remains incompletely understood; however, several genes, including fibrillin-3 (*FBN3*), the gonadotropin-releasing hormone receptor (*GnRHR*), and *CYP1B1*, which is involved in estradiol hydroxylation, have been implicated in the development of both PCOS and HT (45). Polymorphisms in the *FBN3* gene may play a shared role in these conditions, potentially through modulation of transforming growth factor-β (TGF-β) activity. TGF-β is a multifunctional cytokine essential for maintaining immune tolerance by promoting regulatory T-cell function and limiting excessive immune responses. Reduced TGF-β1 levels have been observed in patients with HT and PCOS, particularly in women carrying the D19S884 allele 8 variant of *FBN3*, which may contribute to impaired immune regulation and the increased prevalence of HT among individuals with PCOS (46, 47).

The compensatory hyperinsulinemia stimulates ovarian theca cells to increase androgen production and suppresses hepatic synthesis of sex hormone-binding globulin (SHBG). This results in elevated free androgen levels, a hallmark of PCOS. Hypothyroidism further reduces SHBG levels, amplifying hyperandrogenism and worsening PCOS manifestations such as anovulation and menstrual irregularities (48, 49). Another important factor is leptin dysregulation in the adipose tissue and hypothalamic–pituitary axis dysregulation in hypothyroidism, which may further contribute to the development of PCOS (50).

In the present study, we could not find an association between total cholesterol, LDL, HDL, and triglycerides in patients with PCOS with and without HT. Previous studies showed higher dyslipidemia in patients with PCOS and HT compared to those with PCOS alone (21, 22). A plausible explanation could be the small number of included studies in this meta-analysis.

The strength of this meta-analysis is that we included the largest up-to-date studies, avoided overlap, and excluded poster presentations. In addition, to the best of our knowledge, no researchers have conducted meta-analyses on the effects of HT in patients with PCOS.

### Study limitations

The substantial heterogeneity observed across several pooled analyses likely reflects important methodological and clinical differences among the included studies. Participant age varied considerably, ranging from adolescents to middle-aged women, and the body mass index ranged from normal weight to obesity. Importantly, both age and adiposity are strongly associated with insulin resistance, thyroid autoimmunity, and metabolic outcomes (51, 52). The variation in these characteristics may have contributed to between-study differences. Another important source of heterogeneity is geographic variation; in this meta-analysis, most studies were conducted in Asia, whereas others were published in Europe and South America, where genetic backgrounds, environmental exposures, iodine intake, and healthcare practices differ. Furthermore, studies employed different designs (cross-sectional, case–control, prospective, and retrospective), which may influence effect estimates and susceptibility to bias. Although most studies used the Rotterdam criteria for PCOS diagnosis, differences in HT definitions, thyroid antibody thresholds, thyroid function status, and laboratory measurement techniques may also have contributed to heterogeneity. These factors should be considered when interpreting the pooled estimates. An additional limitation is the broad age range represented by the included studies. Some cohorts enrolled adolescent girls, whereas others included predominantly adult or middle-aged women. The clinical presentation of PCOS and the prevalence of thyroid autoimmunity, insulin resistance, and lipid abnormalities may differ across age groups. Adolescents often have evolving reproductive and metabolic phenotypes, while older women may have accumulated metabolic risk factors and a higher prevalence of autoimmune disease (53, 54). Consequently, pooling these populations may have increased heterogeneity and may limit the generalizability of the findings to specific age groups. Future studies stratified by age category would help clarify whether the association between PCOS and HT differs across the lifespan.

### Clinical implications

The coexistence of HT, insulin resistance, and PCOS has important clinical implications. Women with HT may benefit from screening for insulin resistance and PCOS, particularly in the presence of menstrual irregularities or hyperandrogenic features. Conversely, thyroid function and autoantibodies should be assessed in women with PCOS, as early detection and treatment of thyroid dysfunction may improve metabolic and reproductive outcomes.

### Conclusion

Women with PCOS had significantly higher odds of Hashimoto thyroiditis than controls. Among women with PCOS, the coexistence of HT was associated with higher insulin resistance, whereas lipid parameters did not differ significantly, with no differences in dyslipidemia (TC, LDL, HDL, and TG). Prospective studies with long durations are recommended to investigate the association between PCOS and various diseases.

## Data Availability

The original contributions presented in the study are included in the article/supplementary material. Further inquiries can be directed to the corresponding author.
